# Site and Mechanism of ML252 Inhibition of Kv7 Voltage-Gated Potassium Channels

**DOI:** 10.1093/function/zqad021

**Published:** 2023-05-04

**Authors:** Richard Kanyo, Shawn M Lamothe, Arturo Urrutia, Samuel J Goodchild, W Ted Allison, Richard Dean, Harley T Kurata

**Affiliations:** Dept. of Pharmacology, Alberta Diabetes Institute, University of Alberta, 9-70 Medical Sciences Building, Edmonton, AB T6G 2H7, Canada; Dept. of Pharmacology, Alberta Diabetes Institute, University of Alberta, 9-70 Medical Sciences Building, Edmonton, AB T6G 2H7, Canada; Dept. of Cellular and Molecular Biology, Xenon Pharmaceuticals Inc., 3650 Gilmore Way, Burnaby, BC V5G 4W8, Canada; Dept. of Cellular and Molecular Biology, Xenon Pharmaceuticals Inc., 3650 Gilmore Way, Burnaby, BC V5G 4W8, Canada; Dept. of Biological Sciences, University of Alberta, Edmonton, AB T6G 2E9, Canada; Dept. of Cellular and Molecular Biology, Xenon Pharmaceuticals Inc., 3650 Gilmore Way, Burnaby, BC V5G 4W8, Canada; Dept. of Pharmacology, Alberta Diabetes Institute, University of Alberta, 9-70 Medical Sciences Building, Edmonton, AB T6G 2H7, Canada

**Keywords:** potassium channel, pharmacology, M-current, Kv7 channel, KCNQ channel

## Abstract

Kv7 (KCNQ) voltage-gated potassium channels are critical regulators of neuronal excitability and are candidate targets for development of antiseizure medications. Drug discovery efforts have identified small molecules that modulate channel function and reveal mechanistic insights into Kv7 channel physiological roles. While Kv7 channel activators have therapeutic benefits, inhibitors are useful for understanding channel function and mechanistic validation of candidate drugs. In this study, we reveal the mechanism of a Kv7.2/Kv7.3 inhibitor, ML252. We used docking and electrophysiology to identify critical residues involved in ML252 sensitivity. Most notably, Kv7.2[W236F] or Kv7.3[W265F] mutations strongly attenuate ML252 sensitivity. This tryptophan residue in the pore is also required for sensitivity to certain activators, including retigabine and ML213. We used automated planar patch clamp electrophysiology to assess competitive interactions between ML252 and different Kv7 activator subtypes. A pore-targeted activator (ML213) weakens the inhibitory effects of ML252, whereas a distinct activator subtype (ICA-069673) that targets the voltage sensor does not prevent ML252 inhibition. Using transgenic zebrafish larvae expressing an optical reporter (CaMPARI) to measure neural activity in-vivo, we demonstrate that Kv7 inhibition by ML252 increases neuronal excitability. Consistent with in-vitro data, ML213 suppresses ML252 induced neuronal activity, while the voltage-sensor targeted activator ICA-069673 does not prevent ML252 actions. In summary, this study establishes a binding site and mechanism of action of ML252, classifying this poorly understood drug as a pore-targeted Kv7 channel inhibitor that binds to the same tryptophan residue as commonly used pore-targeted Kv7 activators.

ML213 and ML252 likely have overlapping sites of interaction in the pore Kv7.2 and Kv7.3 channels, resulting in competitive interactions. In contrast, the VSD-targeted activator ICA-069673 does not prevent channel inhibition by ML252.

## Graphical Abstract

**Graphical Abstract:**
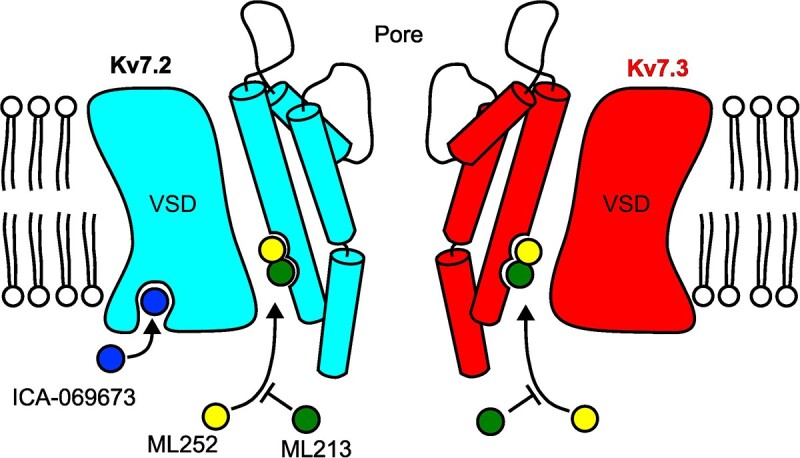

## Introduction

Neuronal Kv7 (KCNQ) voltage-gated potassium channels encode the noninactivating “M-current” that modulates neuronal threshold and firing properties.^1^ The primary subunits involved in generation of M-current in the CNS are Kv7.2 and Kv7.3, although alternative assemblies involving Kv7.4 and Kv7.5 may also contribute.^2,3^ Mutations in each of these subunits (Kv7.2–7.5) have been identified in patients with neurological disorders including benign familial neonatal/infantile seizures, epileptic encephalopathy, and progressive hearing loss.^4–8^

Due to their powerful modulatory role and relevance in disease, neuronal Kv7 channels have been identified as an important therapeutic target, particularly for development of antiseizure medications.^9^ Many small molecules that modulate Kv7.2/Kv7.3 channels have been discovered, with a predominant focus on activator compounds,^10–14^ while far fewer Kv7 inhibitors have been identified.^15,16,17^ Kv7 activators typically shift the voltage-dependence of activation to hyperpolarized voltages by diverse mechanisms, and in some cases increase the maximum macroscopic conductance.^18^ Kv7 activators are divided into at least two categories [pore-targeted or voltage sensing domain (VSD)-targeted] based on their site and mechanism of action.^10^,^19–24^ Both pore- and VSD-targeted subtypes can suppress seizures in animal models, and retigabine (a pore-targeted activator) is the first Kv7 activator to be approved for seizure treatment in humans.^10^,^12–14^,^17^,^25–27^ Pore-targeted activators like retigabine or ML213 require a Trp (Trp236 in Kv7.2, Trp265 in Kv7.3) in the pore-forming S5 segment for efficacy.^18,21,25^ A combination of experimental and structural findings suggest the importance of a H-bond formed between these drugs and the Trp side chain.^28–31^ In contrast, VSD-targeted drugs (eg, ICA-069673, ztz-240, ICA-27243) do not depend on this interaction but are sensitive to mutations in the VSD, and recent cryo-EM structures have highlighted a distinct binding site for ztz-240 in the VSD.^22,27,29^

Endogenous suppression of Kv7 channels is typically mediated by Gq-coupled GPCR signaling that culminates in cleavage of PIP2, leading to increased neuronal excitability.^2,32^ While some small molecule inhibitors of Kv7 channels have been identified and commonly used, these have not been investigated as extensively as Kv7 activators. Recent functional and structural studies have begun to reveal details regarding the mechanism and potential site of action of the most commonly used inhibitors linopirdine and XE991.^15,30^ XE991 and linopirdine have been used to investigate roles of Kv7 channels in epilepsy, long-term potentiation, and neuroplasticity, and are also frequently used to experimentally confirm/validate the mechanism of candidate Kv7 activators.^15^,^33–35^ In addition to XE991 and linopirdine, ML252 has been identified from a high-throughput screen as a Kv7.2/Kv7.3 inhibitor, with stronger potency than XE991,^16^ but its structure is distinct from linopirdine and XE991 and little is known about its molecular mechanism of action. In this study, we report the mechanism of ML252 inhibition of Kv7.2 and Kv7.3, which arises from interaction with a site that likely overlaps the Trp residue required for sensitivity to pore-targeted activators. This feature leads to distinct functional interactions between ML252 and pore- vs. VSD-targeted activator subtypes in-vitro (electrophysiological recordings), and in-vivo (optical monitoring of neuronal activity in zebrafish larvae). These findings establish a clear mechanism of action of a poorly characterized Kv7 inhibitor, and also demonstrate the importance of understanding potential interactions that may arise between inhibitors and activators when validating mechanisms of action of candidate antiseizure medications.

## Method

### Animal Ethics

Husbandry of animals was carried out as per the Canadian Council on Animal Care. The use of Xenopus laevis and Danio rerio (Zebrafish) were approved by the University of Alberta institutional Animal Care and Use Committees under protocols, AU00001752 and AUP00000077, respectively.

### Drug Storage

ML252 was obtained from Sigma-Aldrich. ICA-069673 and ML213 were obtained from Tocris. Stock solutions for manual voltage clamp experiments were prepared in DMSO at 50 m m for ML252 and ML213, and 100 m m for ICA-069673. All stock solutions for automated patch clamp experiments (executed at Xenon Pharmaceuticals) were prepared at 20 m m in DMSO. Working solutions were prepared in extracellular recording solutions at indicated concentrations on each experimental day.

### Xenopus Laevis Oocyte Injections

Kv7.2 and Kv7.3 plasmid constructs, mRNA expression, preparation, and injection of oocytes followed standard procedures that have been described in detail previously.^20^ Briefly, cRNA was synthesized using mMESSAGE mMACHINE SP6 or T7 Transcription Kits (Ambion/Life technologies) using linearized plasmids as templates. Human Kv7.2 and wild-type Kv7.3 constructs were transcribed from a pTLN plasmid via using the Sp6 primer. Homomeric Kv7.3 expression was achieved using the A315T mutation to promote efficient trafficking to the cell surface.^36–38^ Kv7.3[A315T] and Kv7.3[W265F/A315T] were generated in pSRC5. Kv7.1 was generated in pcDNA3.1(−) plasmids and Kv7.5 in pBluescript-II-sk(+). cRNA from pSRC5, pcDNA3.1(−), Pbluescript-II-sk(+) was synthesized using a T7 primer. Oocytes at stages V–VI were selected and injected with cRNA. Homomeric Kv7.3* and heteromeric Kv7.2/Kv7.3 channels were typically recorded within 2 d of injections. Kv7.2 homomeric channels required longer incubations for expression of ideal currents and were typically recorded approximately 72 h after injection.

### Two-Electrode Voltage Clamp Recordings

Two-electrode voltage clamp of *Xenopus* oocytes was performed in a modified Ringer’s solution (116 m m NaCl, 2 m m KCl, 2 m m MgCl2, 1.5 m m CaCl2, and 5 m m HEPES, pH 7.6) using an OC-725C voltage clamp (Warner). Current traces were acquired using a Digidata 1440A (Molecular Devices) and pClamp 10 software (Molecular Devices). Recordings were filtered at 2 kHz and sampled at 5 kHz.

### Manual Patch-Clamp Recordings

Manual patch-clamp recordings with rapid solution exchange were performed using HEK cells transfected with plasmids encoding human Kv7.2 and Kv7.2 [W236F], as previously described.^20^ HEK293 cells were maintained in Dulbecco’s modified eagle medium supplemented with 10% FBS and 1% penicillin/streptomycin. Cells were grown in Falcon tissue culture–treated flasks, in a 5% CO_2_ and 37°C incubator. Cells were seeded into 12-well plates and allowed to settle for 24–48 h before transfection. Cells were transfected using jetPRIME DNA transfection reagent (Polyplus) for 48–72 h prior to experiments. Patch-clamp recordings were obtained in extracellular solution consisting of 135 m m NaCl, 5 m m KCl, 2.8 m m Na acetate, 1 m m CaCl_2_(2H_2_O), 1 m m MgCl2(6H_2_O), and 10 m m HEPES, pH 7.4. Intracellular solution consisted of 135 m m KCl, 5 m m EGTA, and 10 m m HEPES, adjusted to pH 7.3. Extracellular solutions were delivered at room temperature (19°C–21°C) by pressure-driven flow (Automate Inc.), through a multibarreled solution delivery turret, driven by the RSC-200 (Biological) rapid solution exchanger to enable solution jumps. Current traces were obtained with an Axopatch-200B Digidata 1440A and processed by pClamp 10 software.

### Automated High-Throughput Patch-Clamp Recordings

High-throughput patch-clamp recordings were obtained similar to previous descriptions using the Qube 384 automated voltage-clamp platform (Sophion Bioscience A/S, Copenhagen, Denmark),^39–41^ and adherent T-REx HEK293 cells (ThermoFisher), stably transfected with an expression vector containing the full-length cDNA coding for the human Kv7.2, Kv7.3, or Kv7.5 *α*-subunits (in Kv7.2/Kv7.3 or Kv7.3/Kv7.5 cell lines). The human Kv7.2, Kv7.3, and Kv7.5 constructs used correspond to GenBank accession NM_172 107, NM_004 519, and NM_019842NM, respectively. Multihole plates were used and each recording represents the sum of currents (10 sites per well), filtered for minimum seal resistance (>30 MΩ membrane resistance) and minimum current signal size (>2 nA) to minimize contamination of the signal with endogenous currents. Leak subtraction protocols and compensation were not applied.

### CaMPARI Imaging of Neural Activity in Zebrafish

A zebrafish transgenic line (Tg[elavl3: CaMPARI (W391F + V398L)]ua3144) expressing the calcium sensor CaMPARI was generated as previously described, by using the Tol2 vector, pDestTol2-elavl3: CaMPARI (W391F + V398L) (kindly shared by Eric Schreiter, HHMI Janelia).^27,42,43^ CaMPARI undergoes photoconversion (PC) from a green to red emission when high intracellular calcium coincides with illumination by an intense PC light generated by a 405 nm LED array (Loctite). Zebrafish larvae were placed in E3 media [as per Westerfield (2007),^44^ but without methylene blue] and exposed to the PC light for 300 s at a distance of 10 cm from the LED array, in various indicated experimental conditions. PC light was applied 30 min after drug application. During PC, the uncovered petri dish containing 10 mL of E3 media floated in a heat sink with water (room temperature). After PC, larvae were anesthetized in 0.24 mg/mL tricaine methanesulfonate (MS-222, Sigma) and embedded in 2% low-gelling agarose (Sigma #A9045) for imaging.^27^

Images were collected as Z-stacks (4 µm steps) using a 20×/0.8 objective, with a laser-point scanning confocal microscope (Zeiss 700) and are presented as maximum intensity projections. We used Imaris 7.6 (Bitman, Zurich), to identify and analyze the 3-dimensional region of interest corresponding to the hindbrain. Relative neural activity was interpreted by calculating the ratio between the red to green mean fluorescence intensities. Data points were presented as a red/green ratio for each individual fish analyzed. One-way ANOVA and Dunnett’s post-hoc test were performed in GraphPad Prism Software (Version 7, GraphPad, San Diego, CA).

### Molecular Docking

Molecular docking was performed using AutoDock Vina 4.2.6.^45^ Published cryo-EM structures of KCNQ2/Kv7.2 structures in complex with either retigabine (PDB 7CR7) or ztz240 (PDB 7CR4) were used as templates, along with a pdb model of ML252 constructed using Maestro (Schrodinger Inc.). Grid boxes in the Kv7.2 models were chosen to encompass the ion conducting pore along with the pore-forming domain of a single subunit along with its interfaces with neighboring subunits and the voltage sensing domain.

## Results

### ML252 Interacts with the Pore of Kv7.2 and Kv7.3

There is a paucity of knowledge regarding the mechanism of action of Kv7 inhibitors and their interactions with therapeutically relevant channel activators. Current inhibitors such as XE991 have some drawbacks as their subtype specificity is unclear and the drug onset and washout is reported to be slow or irreversible.^15^ We investigated ML252, a recently identified Kv7 inhibitor with a previously undescribed site and mechanism of action.^16^

**Figure 1.**
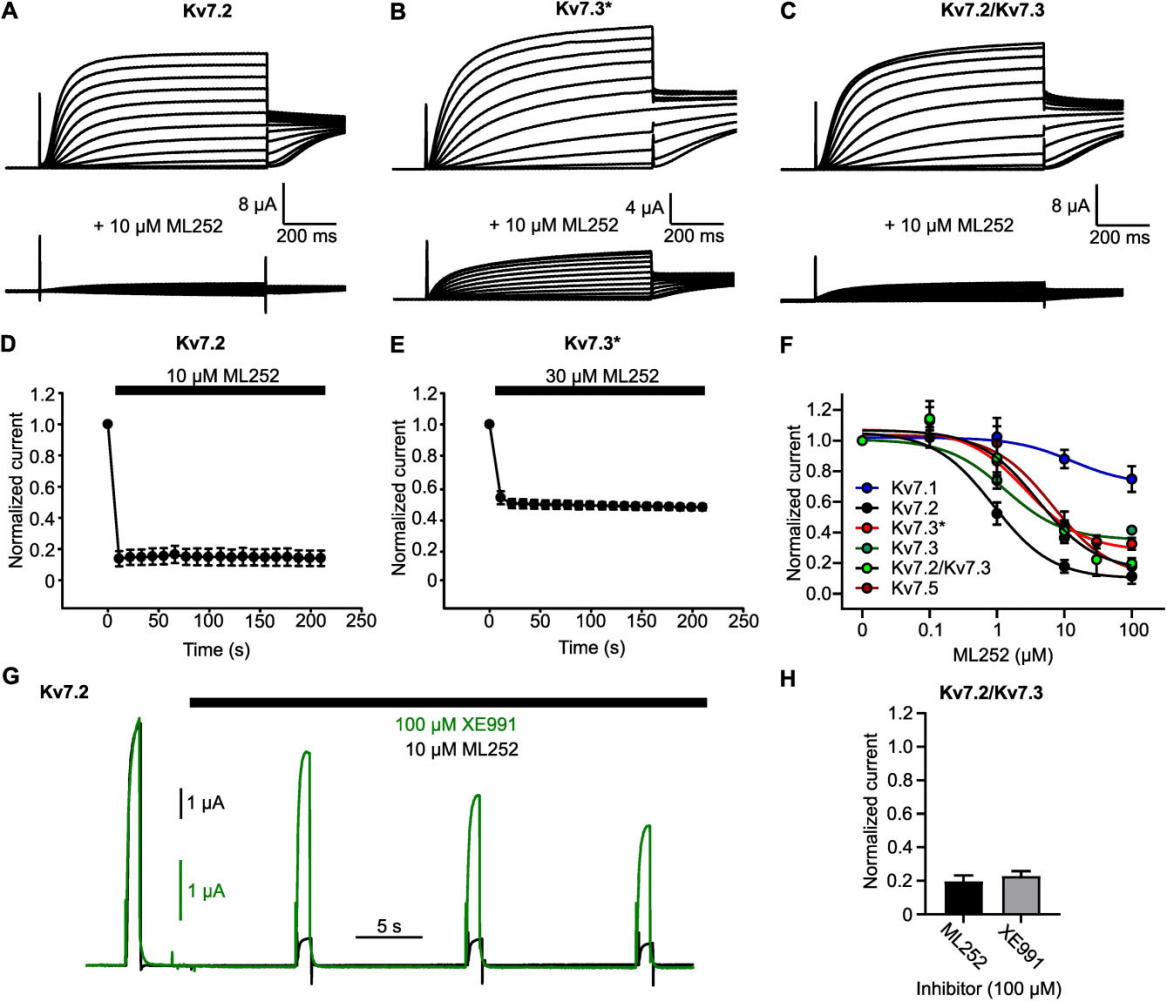
ML252 inhibits Kv7.2 and Kv7.3 homomeric channels with rapid onset. (A to C) Exemplar records from *Xenopus* oocytes expressing indicated channel types, in (top) 0 µm or (bottom) 10 µm ML252. Oocytes were held at −80 mV and pulsed between −140 and +40 mV in 10 mV steps. (D and E) Oocytes expressing (D) Kv7.2 (*n* = 7) or (E) Kv7.3* (*n* = 9) were pulsed from −80 mV to +20 mV for 1 s at 0.1 Hz, and ML252 was applied after the first pulse. (F) Concentration response for ML252 measured at +20 mV from oocytes injected with Kv7.2 (*n* = 4–13; IC50 = 0.88 μm), Kv7.3 [A315T] (Kv7.3*; *n* = 8–13; IC50 = 2.71 μm), WT Kv7.3 (Kv7.3; *n* = 4–8; IC50 = 1.32 μm), Kv7.2/Kv7.3 (*n* = 3–11; IC50 = 4.05 μm), and Kv7.5 (*n* = 5–6; IC50 = 6.70 μm). (G) ML252 (black, 10 μm) or XE991 (green, 100 μm) were applied after the first pulse as shown. Oocytes expressing Kv7.2 were pulsed for 1 s at +20 mV, from a holding potential of −80 mV (0.1 Hz). (H) Maximal inhibition of Kv7.2/Kv7.3 heteromers in 100 μm ML252 (black) or XE991 (gray).

We first used TEVC recordings from *X. laevis* oocytes to assess ML252 inhibition of various Kv7 channels (Figure 1). Kv7.2, Kv7.3 [A315T] (referred to as Kv7.3*), WT Kv7.3, Kv7.2/7.3 heteromers, and Kv7.5 channels were clearly inhibited by ML252 with Kv7.2 channels exhibiting slightly stronger potency and more complete block compared to other Kv7 channel types (Figure 1A–C and F). Kv7.2/7.3 heteromers also exhibited more complete ML252 block than WT Kv7.3 or Kv7.3* channels. In contrast, Kv7.1 was only weakly inhibited by ML252 (Figure 1F), which may be noteworthy as prior descriptions of ML252 reported Kv7.1 inhibition with appreciable potency (∼2 μm) but did not highlight the weak efficacy toward Kv7.1.^16^ To investigate the onset of ML252 inhibition, we pulsed channels to +20 mV (1 s pulses, 0.1 Hz) during drug wash-in (Figure 1D and E) and observed that ML252 effects have a rapid onset, with complete block typically observed after the first pulse during drug wash-in. This rapid onset of ML252 contrasts with the previously described slow onset of XE991 and linopirdine, which exhibit use-dependence and require prolonged channel activation for significant channel block to occur.^15,46^ We confirmed this finding, shown by exemplar traces in Figure 1G in which oocytes expressing Kv7.2 were exposed to ML252 or XE991. ML252-mediated inhibition was complete after 1 pulse, whereas XE991 was only partially effective over these short applications. Although these findings do not explicitly measure rates of ML252 binding to closed vs. open channels, they illustrate much faster inhibition by ML252 and suggest that unlike XE991, ML252 can bind to closed channels during the interpulse interval. If channels are exposed to the drugs for a sufficient duration, the extent of the block of heteromeric Kv7.2/Kv7.3 channels is similar for ML252 and XE991 in our experimental system (Figure 1H). We also compared XE991 and ML252 inhibition in HEK cells using an automated planar patch clamp approach. Both drugs caused similar maximal inhibition of Kv7.2/Kv7.3 or Kv7.3/Kv7.5, but ML252 exhibited more potent inhibition of Kv7.3/Kv7.5 heteromeric channels (Figure S5).

**Figure 2.**
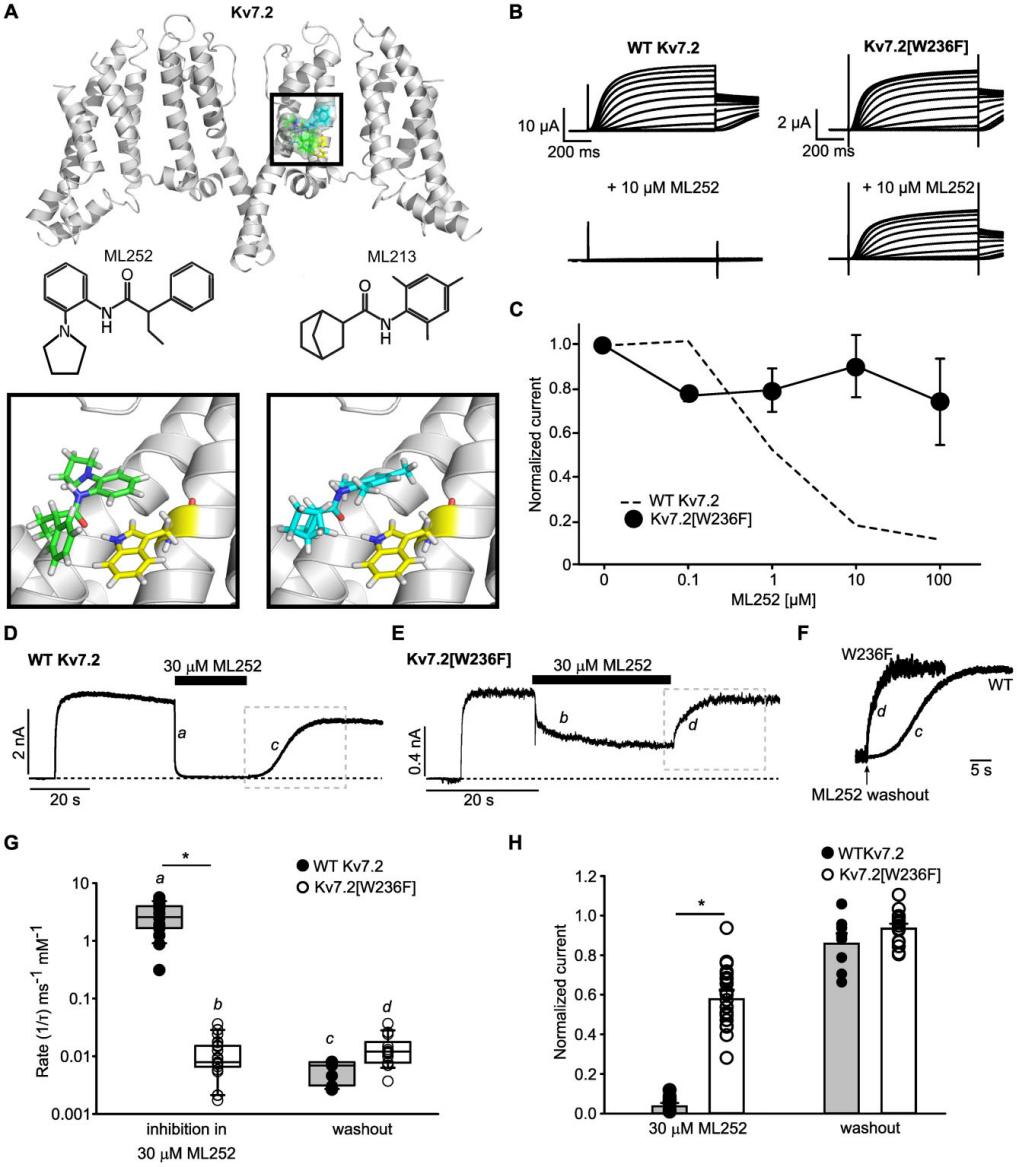
Kv7.2 Trp236 strongly influences ML252 inhibition. (A) Docked poses of ML252 (green) in the cryo-EM structure of Kv7.2 (PDB 7VNP), along with the experimentally reported binding mode of ML213 (cyan). The binding pocket is enlarged in the inset, illustrating the orientation of ML252 (left) or ML213 (right) relative to Trp236 (yellow). (B) Exemplar traces from *Xenopus* oocytes expressing WT Kv7.2 (left) or Kv7.2[W236F] (right), in control or 10 μm ML252, as indicated. Voltage was held at −80 mV and pulsed between −140 and +40 mV (10 mV steps). (C) ML252 concentration response of Kv7.2[W236F] in *Xenopus* oocytes at +20 mV (*n* = 5) (WT Kv7.2 data from Figure 1F re-plotted as dashed line). (D and E) Exemplar patch-clamp current recordings of (D) WT Kv7.2 or (E) Kv7.2[W236F] expressed in HEK293 cells. Cells were depolarized and held at +20 mV, followed by drug application or washout where indicated. Segments *a* and *b* were used to measure the rate of onset of inhibition, segments *c* and *d* were used to measure the rate of recovery. (F) Expanded view of the kinetics of current recovery after ML252 washout in WT Kv7.2 or Kv7.2[W236F] (boxes *c* and *d* from panels D and E). (F and G) Traces obtained from ML252 washouts expressing WT Kv7.2 were fit with a Hodgkin–Huxley equation and Kv7.2[W236F] traces were fit with a single exponential function. (G) Rates of ML252 inhibition and recovery in WT Kv7.2 (recovery *n* = 11, inhibition *n* = 20) or Kv7.2[W236F] (recovery *n* = 17, inhibition *n* = 28) (* indicates *P* < .001, Student’s *t*-test). (H) Current magnitudes (normalized to maximal current at +20 mV) in ML252 or after washout, for WT Kv7.2 (*n* = 12 in 30 μm ML252, *n* = 8 washout) or Kv7.2[W236F] (*n* = 25 in 30 μm ML252, *n* = 15 washout), (* indicates *P* < .001, Student’s *t*-test of current inhibition of WT 7.2 vs. 7.2[W236F]).

To investigate the location of ML252 binding to Kv7 channels, we docked the compound into a recently published structure of Kv7.2.^29^ We were especially interested in drug poses near the region of the retigabine binding site (Kv7.2 Trp236), as our findings (Figure 1F) along with previous reports indicate that ML252 causes weaker inhibition of Kv7.1 relative to other Kv7 channels, and this position contributes to differential sensitivity of Kv7.1 to retigabine and other pore-targeted activators.^1,2,16^ A favorable pose of ML252 in this site is shown in Figure 2A, and features orientation of the ML252 carbonyl toward Kv7.2 Trp236 in a potential H-bond interaction. This interaction is similar to the H-bond interaction between ML213 (or retigabine) and Trp236, suggested by functional studies and recent cryo-EM structures. Therefore, we investigated the functional contribution of Kv7.2 Trp236 (and Kv7.3 Trp265) to ML252 sensitivity, using TEVC and patch clamp recordings. Kv7.2[W236F] exhibited markedly reduced ML252 sensitivity relative to WT Kv7.2 (Figure 2B and C). Attenuated ML252 inhibition was also observed in Kv7.3*[W265F] (equivalent to Kv7.2[W236F], Figure S1). We also used fast solution switching with patch-clamp recordings of transfected HEK293 cells to assess the kinetics and sensitivity of ML252 inhibition. In these HEK293 recordings, Kv7.2[W236F] was also resistant to ML252 (Figure 2D to H). ML252 (30 μm) generated nearly complete inhibition of WT Kv7.2 with a rapid onset (Figure 2D, G, and H), whereas Kv7.2[W236F] channels exhibited slower and incomplete block (Figure 2E, G, and H). In these experiments, it is noteworthy that the Trp236Phe mutation did not completely abolish ML252 sensitivity, as there were variable levels of ML252 inhibition of Kv7.2[W236F] (Figure 2E and H). This observation may indicate the presence of lower affinity sites for ML252 inhibition (either in the mutated retigabine binding site, or elsewhere in the channel), as the concentration used in these rapid perfusion experiments is high relative to the ML252 IC50 determined in Figure 1 (0.88 μm), although we have not explored this further. Kv7.2 channels also exhibit variable degrees of rundown in HEK293 cells and this may contribute to the variability of ML252 inhibition.

Interestingly, ML252 washout from WT Kv7.2 channels exhibited a prominent delay leading to sigmoidal washout kinetics, whereas washout from Kv7.2[W236F] channels was monoexponential and faster (Figure 2F, inset). This finding may reflect the lower affinity modes of ML252 binding stated above and could imply that multiple ML252 unbinding events are required for relief of inhibition of WT Kv7.2 channels. Regardless, considering the large impact of mutating Trp236, these results suggest that the Trp236 in Kv7.2 (or Trp265 in Kv7.3), is critical for ML252 inhibition in addition to its established role in sensitivity to pore-targeted activators. These findings also provide an explanation for the enhanced ML252 sensitivity of Kv7.2 and Kv7.3 relative to Kv7.1 (which has a Leu at the pore position equivalent to Kv7.2[Trp236], Figure 1F).^16,18,47^

### Competition Between ML252 and Pore-Targeted Activators

**Figure 3.**
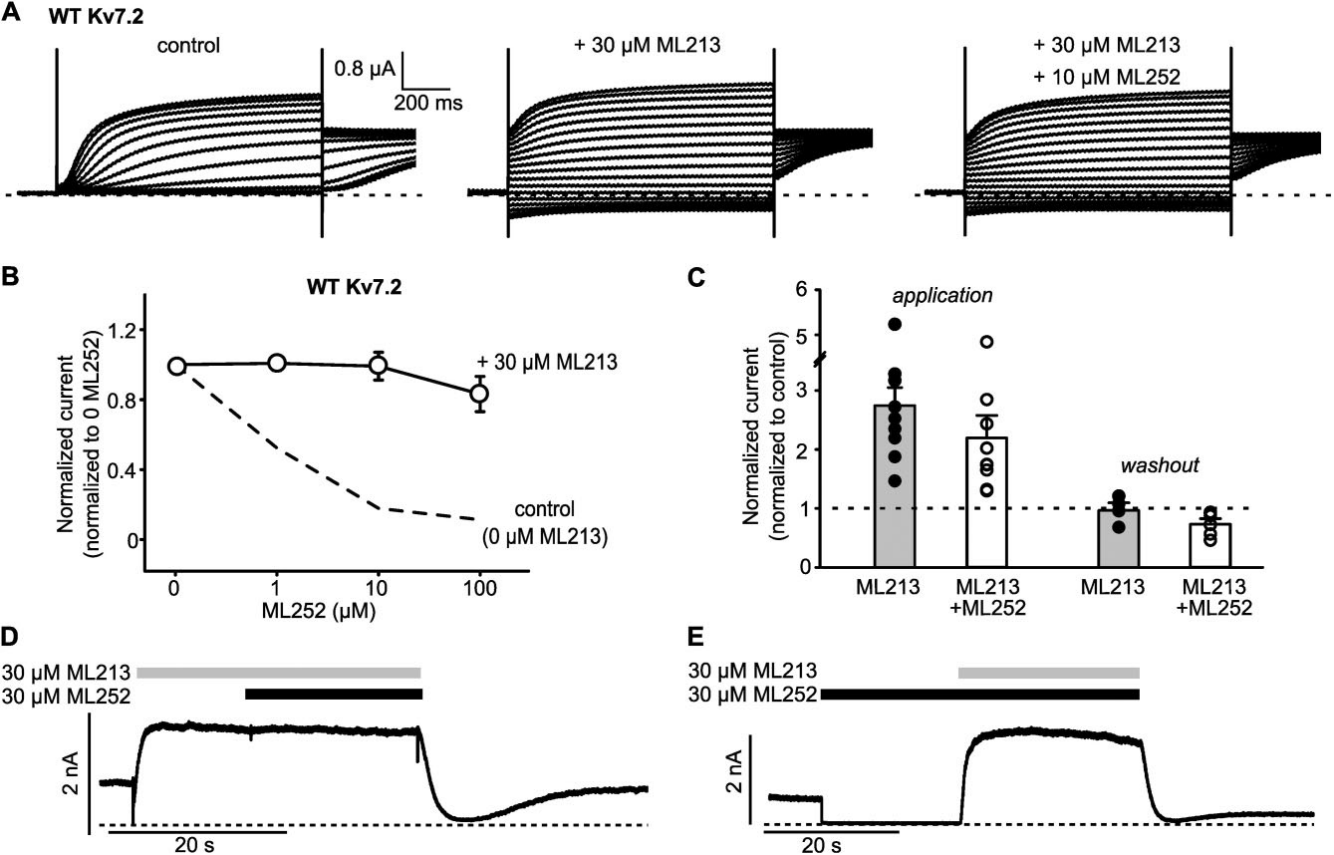
The pore-targeted activator ML213 weakens ML252 inhibition of Kv7.2. (A) Exemplar records of Kv7.2 expressed in *Xenopus* oocytes, treated with ML213 and ML252 as indicated. Oocytes were held at −80 mV and pulsed between −140 and +40 mV in 10 mV steps. (B) WT Kv7.2 concentration response to ML252, collected in the presence (open circles, *n* = 4–6) or absence (dashed line, data from Figure 1F) of ML213. Currents were measured at +20 mV and normalized to current in 0 μm ML252. (C to E) Fast-perfusion patch-clamp recordings using HEK cells showing ML213 competition with ML252. (C) Normalized Kv7.2 current magnitudes from HEK293 cells upon application or washout with indicated combinations of 30 μm ML213 and 30 μm ML252, normalized to currents measured in the absence of drugs and collected as depicted in panels D and E (application, *n* = 9 for ML213, *n* = 9 for ML213 + ML252) (washout, *n* = 4 for ML213, *n* = 6 for ML213 + ML252). No statistical difference was found for inhibition by ML213 vs. ML213 + ML252 (*P* = .122, Student’s t-test). (D and E) Exemplar patch-clamp recordings of HEK293 cells expressing WT Kv7.2. Cells were held at +20 mV throughout the recording and combinations of ML252 and ML213 were applied where indicated.

Based on the potential overlap of binding sites for ML252 and other pore-targeted activators, we tested the functional relationship between ML252 and a pore-targeted activator, ML213 (Figure 3). Application of ML213 enhanced Kv7.2 channel activation as demonstrated by conductance of currents at negative voltages (Figure 3A, middle), due to a hyperpolarizing shift of the voltage-dependence of activation previously described for this drug.^27,28^ However, application of ML252 (10 μm) in combination with ML213, failed to inhibit channels (Figure 3A and B, recordings from oocytes). This observation suggested that ML252 interaction with Trp236 could be prevented by ML213, consistent with the idea that these compounds compete for binding to the modulatory pore site (rather than ML252 acting as a pore blocker). Further experiments in oocytes demonstrated that ML252 inhibition of Kv7.3* (Figure S2) or Kv7.2 (Figure S3) was attenuated by ML213. Moreover, this functional interaction was also observed if drugs were applied sequentially, irrespective of the order of application. This is illustrated using rapid perfusion switches in patch clamp of HEK cells held at +20 mV (Figure 3C to E), in which ML252 application either after (Figure 3D) or before (Figure 3E) ML213 led to similar outcomes on potentiation of current. Similar observations were made using oocytes expressing either Kv7.3* (Figure S2C) or Kv7.2 (Figure S3A). In these experiments ML252 application to channels “pre-activated” with ML213 did not suppress current (Figures S2C and S3A). Similarly, if channels were first inhibited by ML252, subsequent ML213 application led to rapid rescue of currents (Figures S2D and S3B). These results suggest a functional interaction between ML213 and ML252, likely arising from competition for association with Kv7.2 Trp236 (of Kv7.3 Trp265).

### VSD-Targeted Activators Do Not Influence ML252 Inhibition

**Figure 4.**
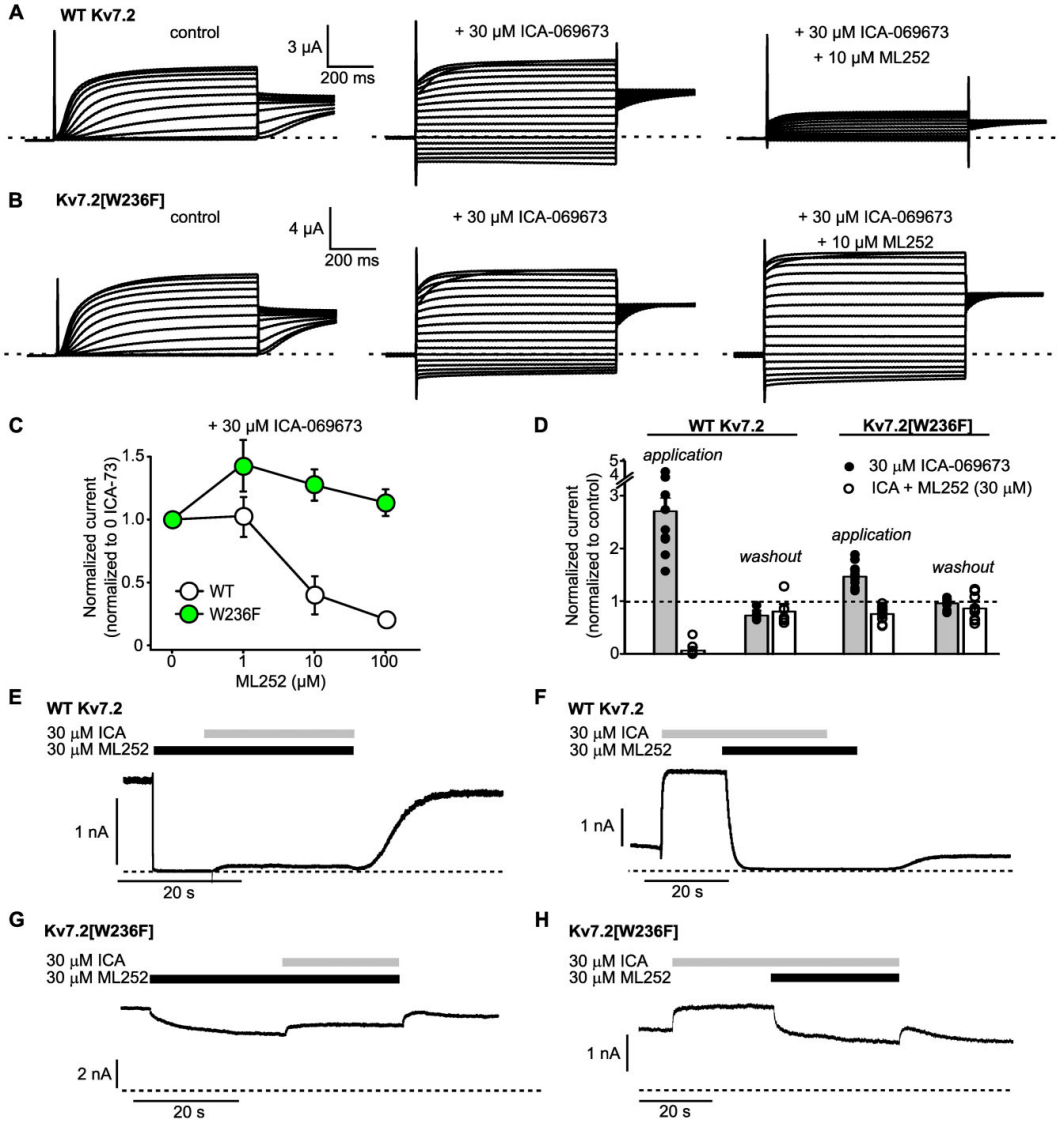
The VSD-targeted activator ICA-069673 does not weaken ML252 inhibition. (A and B) Exemplar records of WT Kv7.2 or Kv7.2[W236F] homomers expressed in *Xenopus* oocytes, and treated with ICA-069673 (ICA) and ML252 as indicated. Oocytes were held at –80 mV and pulsed between –140 and +40 mV in 10 mV steps. (C) ML252 concentration responses were assessed in WT Kv7.2 and Kv7.2[W236F] channels, in the presence of 30 μm ICA-069673. Currents were recorded at +20 mV and normalized to peak current at +20 mV in ICA-069673 alone (*n* = 4–7). (D) Normalized WT Kv7.2 and Kv7.2[W236F] current magnitudes in HEK293 cells upon application or washout with indicated combinations of 30 μm ICA-069673 and 30 μm ML252, normalized to currents measured in the absence of drugs and collected as depicted in panels E to H (For WT Kv7.2: ICA-069673 application, *n* = 9, washout, *n* = 5; ICA-069673 + ML252 application, *n* = 12, washout, *n* = 6) (For Kv7.2[W236F]: ICA-069673 application, *n* = 12, washout, *n* = 8; ICA-069673 + ML252 application, *n* = 12, washout, *n* = 9 washout of ICA-069673 and ML252). (E to H) Exemplar patch-clamp recordings of HEK293 cells expressing WT Kv7.2 (E and F) or Kv7.2[W236F] (G and H). Cells were held at +20 mV throughout the recording and combinations of ML252 and ICA-069673 were applied where indicated, dashed lines indicate zero current.

Activators of Kv7.2/Kv7.3 channels can be categorized based on their association with either the pore (eg, retigabine and ML213) or the VSD (eg, ICA-069673, ICA-27243, ztz-240).^10^,^19–24^ To further investigate functional interactions of ML252 with activator compounds, we performed similar experiments testing combinations of ICA-069673 (Kv7.2 VSD-targeted) and ML252. Kv7.2 homomers exhibited concentration-dependent inhibition by ML252 even with co-application of 30 μm of ICA-069673 (Figure 4A and C), whereas Kv7.2[W236F] channels were insensitive to ML252 in the presence of ICA-069673. This again confirmed the importance of the pore Trp for ML252 sensitivity, and suggested that drugs targeting the VSD do not markedly impair ML252 inhibition (Figure 4B and C). Sequential co-application of ICA-069673 and ML252 in oocyte or HEK cell systems demonstrated that ICA-069673 did not prevent ML252 inhibition (Figure 4D and F; Figure S3C). Carried out in reverse order, ICA-069673 could not rescue channels from ML252 inhibition (Figure 4E; Figure S3D), in contrast to the effects of ML213 described in Figure 3. A consistent explanation for the different functional interactions ML213 or ICA-069673 is that these activators occupy different binding sites, and only ML213 is able to prevent binding and inhibition by ML252 in the pore site.

**Figure 5.**
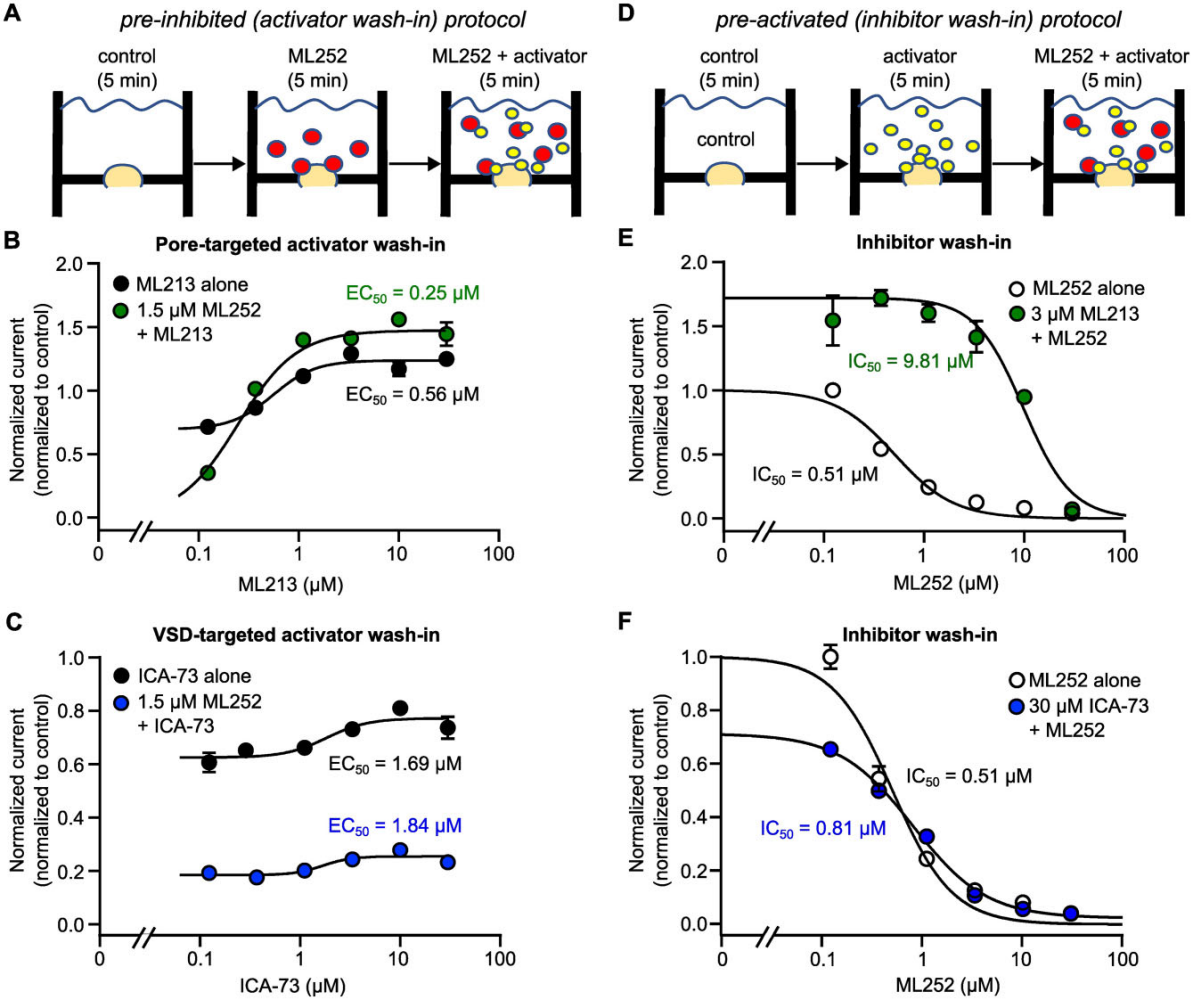
Automated patch clamp analysis of ML252 interactions with Kv7 activators in Kv7.2/Kv7.3 heteromeric channels. (A and D) Drug perfusion paradigms to assess functional outcomes of interactions between ML252 and either ML213 or ICA-069673 (ICA-73). In (A), currents were first recorded in control (no drugs added), following a 5-min incubation in ML252 (at ∼IC50 as determined in Figure S4A), and finally after a combination of either activator (multiple concentrations) with ML252. Currents were measured at a pulse to +20 mV where channels are maximally activated, from a holding potential of −80 mV. In (D), currents were first recorded in control (no drugs added), following a 5-min incubation in ICA-73 (30 μm) or ML213 (3 μm), and finally after a combination of either activator with ML252 (multiple concentrations). (B and C) Concentration responses of Kv7.2/Kv7.3 channels to (B) ML213 or (C) ICA-73, administered alone or after preincubation in 1.5 μm ML252. (E and F) Concentration responses of Kv7.2/Kv7.3 to ML252 alone or after preincubation with (E) ML213 or (F) ICA-73. In panels B, C, E, and F, currents (measured at +20 mV) were normalized to response in control conditions in each respective wells containing up to 10 cells (*n* = 7–8 wells).

We recognized that the drugs tested have different affinities and this may have impacted our findings in experiments conducted at single concentrations of the individual drugs. In order to test our findings over wider concentration ranges, and better characterize the functional interactions of ML252 with pore- or VSD-targeted activators, we used automated patch clamp of HEK cells stably expressing Kv7.2/Kv7.3 (Figure 5). We established an IC50 for ML252 inhibition (1.42 μm) and EC50 (for potentiation of current at +20 mV) by either ML213 (109 n m) or ICA-069673 (380 n m) in the Kv7.2/Kv7.3 cell line used for these experiments (Figure S4A and B). Next, using a constant ML252 concentration (1.5 μm, near the IC50), we tested effects of a wide concentration range of either Kv7 activator compound (Figure 5A to C). Consistent with findings from manual electrophysiology approaches, ML213 potentiation of current at +20 mV was retained in the presence of ML252, and ML213 concentrations above 300 n m largely overcame ML252-mediated inhibition of Kv7.2/Kv7.3 (Figure 5B). In contrast, Kv7.2/Kv7.3 channels were inhibited by ML252 across the entire range of ICA-069673 concentrations tested (Figure 5C), indicating that ICA-069673 could not overcome ML252-mediated inhibition even when the activator was applied at a much higher concentration. We used a similar approach to determine the effects of ML213 and ICA-069673 on the concentration response of ML252. For this experiment, we first applied ML213 (3 µm) or ICA-069673 at an even higher concentration (30 µm), followed by application of various ML252 concentrations (Figure 5D). ML213-mediated potentiation was unaffected by ML252 concentrations ≤ 10 µm, and shifted the ML252 concentration response to ∼30-fold higher concentrations, indicative of competitive displacement (Figure 5E). In contrast, the concentration-dependence of ML252 was not prominently affected by ICA-069673, indicating that ML252 inhibition is not impeded by ICA-069673, even at high concentrations of the activator (Figure 5F). Another reflection of the functional interaction between ML213 and ML252 arose from effects on voltage-dependence of activation. ML213 causes a prominent hyperpolarizing shift of the conductance–voltage relationship. However, this shift is attenuated when ML213 is co-applied with ML252 (Figure S4D). In contrast, the GV shift mediated mediated by ICA-069673 is not attenuated by ML252 (Figure S4C).

### In-Vivo Validation of ML252-mediated Kv7.2/Kv7.3 Inhibition

We tested ML252 inhibition and its interaction with activator drugs in-vivo, using zebrafish larvae. We used a previously described transgenic zebrafish line expressing a calcium sensor,^27,42,43^ CaMPARI, driven by a pan-neuronal promoter for CNS-restricted expression. CaMPARI undergoes photo-switching by 405 nm light applied in the presence of high calcium, converting from green emission to red emission. The red to green ratio reflects neural activity during the PC period, and can be manipulated by application of pro or anticonvulsant drugs.^27,48,49^

**Figure 6.**
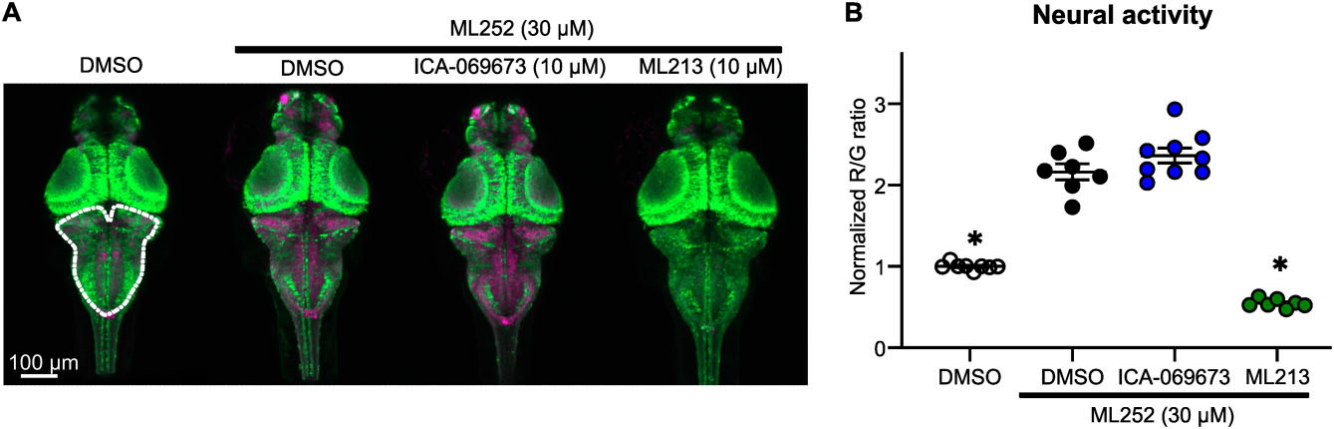
ML252 modifies neuronal activity in zebrafish larvae, and is selectively weakened by the pore-targeted activator ML213. (A) Exemplar images of zebrafish larvae (dorsal view) with neuronal-specific expression of CaMPARI. PC (leading to a higher magenta signal) indicates higher neuronal activity, whereas the absence of PC (weaker magenta signal) indicates low neuronal activity. (B) Summary of PC (red/green ratio) observed in zebrafish larvae treated with ML252 alone or in combination with either ML213 or ICA-069673. Intensities of red (R) and green (G) fluorescence emissions are displayed as red to green ratio and normalized to DMSO controls (quantified in the hindbrain area, dashed white line in panel A) (*n* = 7–9 larvae per condition). * indicates statistical significance vs. ML252 alone (ANOVA followed by Dunn’s post-hoc test, *P* < .01).

We tested the effects of various combinations of ML252, ICA-069673, and ML213 in a CaMPARI photoconversion assay (Figure 6). ML252 (30 μm) increased neural activity measured in the hindbrain area, likely reflecting enhanced excitability caused by Kv7 inhibition (Figure 6A). Co-application with ML213 attenuated ML252-mediated effects on neuronal activity (red:green ratio, Figure 6B). In contrast, co-application of ICA-069673 did not prevent ML252 effects (Figure 6). Consistent with in-vitro electrophysiological findings, these data indicate that a functional interaction between ML213 and ML252 can attenuate ML252-mediated inhibition, whereas ML252 remains effective in the presence of the VSD-targeted activator ICA-069673.

## Discussion

Screening and characterization of the pharmacology of Kv7 channels has primarily focused on activator compounds like retigabine, which have therapeutic benefits for treatment of epilepsy and other neurological disorders.^49,50^ Kv7 inhibitors have been less studied in terms of basic mechanism or biological outcomes, although the most widely used Kv7 inhibitors have been investigated in the context of cognitive enhancement, Parkinson’s disease, and neuroplasticity.^34,35^ These compounds are also frequently used as experimental tools to validate the molecular target of activators (by antagonizing the effects of a candidate activator). Recent strides have been made in terms of understanding the mechanism of action of XE991 and linopirdine, including the demonstration that these drugs exhibit state-dependent inhibition, requiring prolonged or repetitive activation of Kv7 channels for drug onset to occur.^15^ Also, a potential binding site of linopirdine near the intracellular gate of Kv7.4 was identified using cryo-EM, although it is noteworthy that prior functional work indicates an extracellular site of action of linopirdine, suggesting that our understanding of these classically used Kv7 inhibitors remains incomplete.^30,51^

ML252 is a more recently identified Kv7 inhibitor, and thus it has been less frequently used, and there have been few prior insights into its underlying mechanism.^16^ Our study has revealed a likely site and mechanism of action of ML252, along with implications related to its experimental use for validating candidate Kv7 activators. Computational approaches, along with functional experiments with mutant channels (Figures 1 and 2), suggest that ML252 inhibition involves interaction with the pore Trp (Kv7.2 Trp236, Kv7.3 Trp265) required for sensitivity to pore-targeted activator drugs. There is general consensus regarding the likely importance of an H-bond interaction between the Kv7.2 Trp236 indole N-H and carbonyl/carbamate moieties in drugs including ML213 and retigabine.^28,29,31^ Similarly, ML252 comprises a carbonyl that is predicted to interact with the Trp236 side chain, but other features of the drug must cause it to act as an inhibitor rather than an activator. This is consistent with the initial characterization of ML252,^16^ which reported that a small change of an ethyl group to a hydrogen or a fluorine switched the effects of ML252 from inhibition to potentiation. Similarly, small modifications to activator drugs like retigabine have been reported to switch their effects to inhibition.^50^ Overall, ML252 appears to occupy a region within or overlapping the modulatory site that accommodates pore-targeted activator drugs, but with a distinct functional outcome. Thus, our findings suggest that ML252 inhibition is caused by an allosteric effect on gating, rather than direct occlusion of the channel pore.

**Figure 7.**
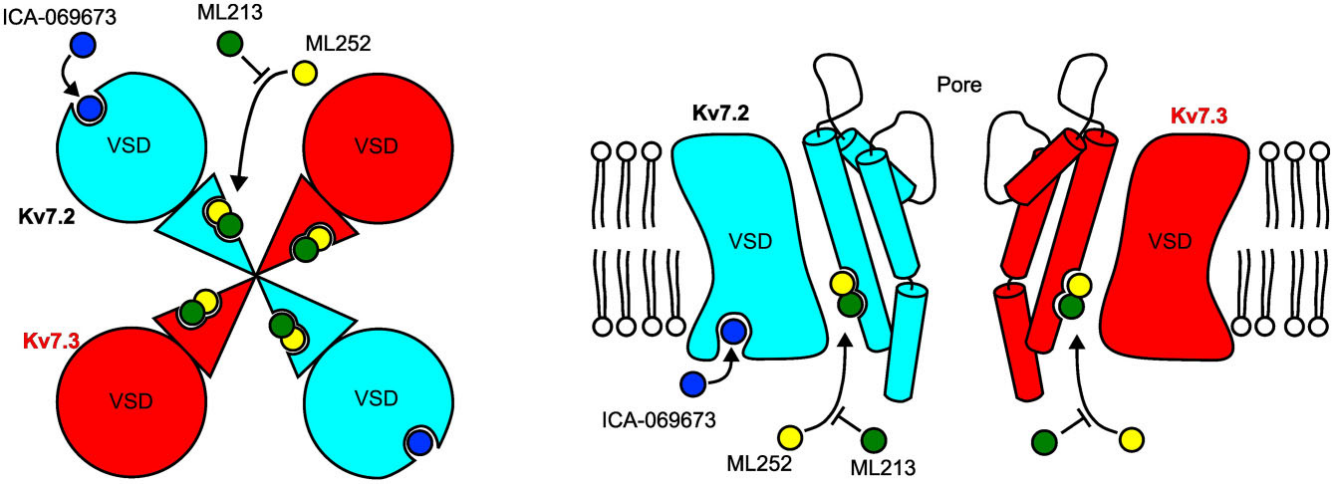
Summary of ML252 inhibition in the context of Kv7 activator binding sites. Mutagenesis and docking suggest that ML252 binds to a similar site as pore-targeted activators including retigabine and ML213 in both Kv7.2 (cyan) and Kv7.3 (red). This underlies less potent ML252-mediated inhibition when applied together with a pore-targeted activator. VSD-targeted activators ztz-240 and ICA-069673 bind to a distinct site (only present in Kv7.2), and therefore do not prevent ML252 inhibition.

The overlapping site of action of ML252 and pore-targeted activators leads to distinct functional outcomes when inhibitors and activators are applied in combination. In-vitro (Figures 3 –5) and in-vivo (Figure 6) studies illustrated that pore-targeted activators can effectively counteract ML252 inhibition, likely due to competitive displacement of the inhibitor at the overlapping binding site (Figure 7). In contrast, the VSD-targeted activator ICA-069673 did not compete with the inhibitor (Figure 7). In the context of the growing understanding of the complex pharmacology of Kv7 activators, these distinct outcomes are consistent with functional and structural evidence for multiple sites of action of pore- vs. VSD-targeted activator drugs.^22,23,29,30,52^ It is now clear that there are multiple potential mechanisms that can be exploited in the design of Kv7 activators, either by trapping the activated VSD conformation, or stabilizing the open pore conformation. In terms of practical applications of ML252 vs. XE991 or linopirdine for mechanistic validation of activator compounds, the recognition that ML252 will have distinct interactions with pore- or VSD-targeted activators is an important consideration for experimental design.

In comparison to the more widely used inhibitors linopirdine and XE991, ML252 has certain benefits. Firstly, ML252 inhibition and washout is rapid and likely binds to both open and closed states of the channel (Figures 1 and 2). In contrast, XE991 and linopirdine exhibit use-dependence and require prolonged channel activation or repetitive stimulation for the block to set in, and washout of these drugs is also slow and often incomplete.^15^ A second benefit of ML252 is that it is effective toward most Kv7 channels including Kv7.2/Kv7.3, and Kv7.3/Kv7.5 (Figures 1; Figure S5), and has been previously used to inhibit neuronal channels, ^53^ but only weakly blocks Kv7.1. In contrast, XE991 and linopirdine inhibit Kv7.1 expressed in cardiomyocytes at concentrations <1 μm (although this is reported to be weaker when Kv7.1 is associated with modulatory KCNE subunits).^16,54^ However, these benefits should be balanced with the aforementioned potential for interactions if ML252 is planned to be used in combination with a Kv7 activator, as is often done in target validation experiments. That is, ML252 can antagonize a pore-targeted activator, but our findings show that this requires much higher concentrations than required if applied alone, and this could be problematic in terms of delivering drugs in an in-vivo model. In contrast, ML252 appears to effectively antagonize the effects of a VSD-targeted activator. Overall, understanding the mechanism of action of a potential activator drug may influence the choice and concentration of inhibitors used in combination for target validation or other applications.

In summary, our work reveals detailed features of the site and mechanism of ML252 inhibition of Kv7.2/Kv7.3 channels. ML252 has a rapid onset and targets a modulatory site that is also affected by pore-targeted Kv7 activators. ML252 sensitivity requires a conserved Trp residue that interacts with pore-targeted activators, and this leads to distinct functional outcomes when ML252 is co-applied with pore- vs. VSD-targeted activator drugs. Overall, these findings highlight the effectiveness of ML252 as an inhibitor of Kv7.2 and Kv7.3, and solidify a growing body of evidence distinguishing Kv7 modulators based on sites of action in the pore or VSD.

## Supplementary Material

zqad021_Supplemental_File

## Data Availability

Data presented in this article were generated in the authors’ laboratories and can be made available upon request.
