# Supplementary material for: Site and Mechanism of ML252 Inhibition of Kv7 Voltage-Gated Potassium Channels
Source: Function (Oxf). 2023 May 4;4(4):zqad021. doi: 10.1093/function/zqad021 (PMC10278987; doi:10.1093/function/zqad021)
Supplement: zqad021_Supplemental_File [file zqad021_supplemental_file.docx]

**Supplementary information for:**

**Site and mechanism of ML252 inhibition of Kv7 voltage-gated potassium channels**

Richard Kanyo^1^, Shawn M. Lamothe^1^, Arturo Urrutia^2^, Samuel J. Goodchild^2^, W. Ted Allison^3^, Richard Dean^2^, Harley T. Kurata^1^

*^1^Dept. of Pharmacology, Alberta Diabetes Institute, University of Alberta,* [*9-70 Medical Sciences Building*](http://www.campusmap.ualberta.ca/index.cfm?campus=1&sector=5&feature=80)*, Edmonton, AB, T6G 2H7, Canada*

*^2^Dept. of Cellular and Molecular Biology, Xenon Pharmaceuticals Inc., 3650 Gilmore Way, Burnaby, BC, V5G 4W8, Canada*

*^3^Dept. of Biological Sciences, University of Alberta, Edmonton, AB, Canada*

Running title: ML252 inhibition of KCNQ channels

Keywords: potassium channel, pharmacology, M-current, Kv7 channel, KCNQ channel

**Corresponding author:** Harley T. Kurata, Current address: Dept. of Pharmacology, Alberta Diabetes Institute, University of Alberta,9-70 Medical Sciences Building, Edmonton, AB, T6G 2H7, Canada, kurata@ualberta.ca


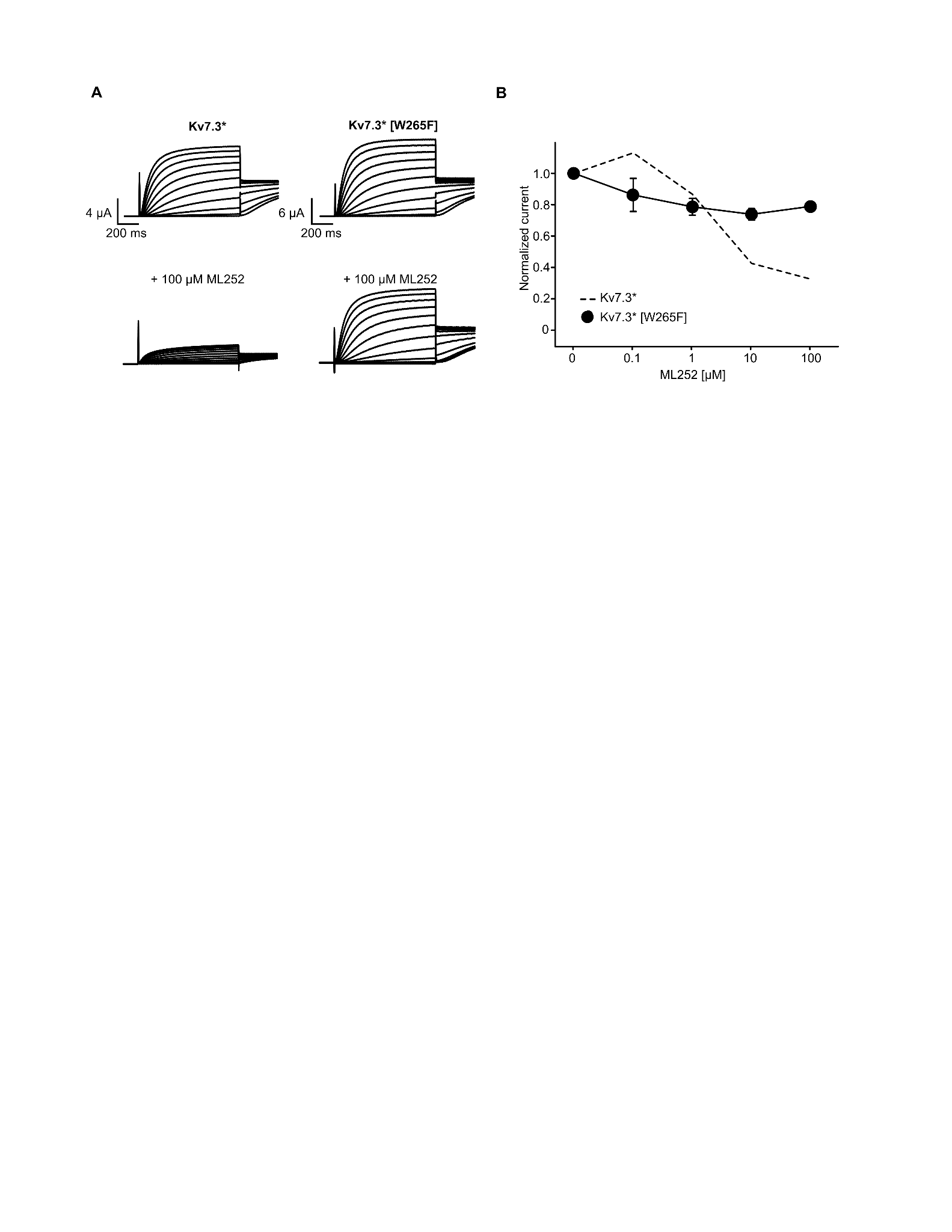


**Supplementary Figure 1: Kv7.3 Trp265 strongly influences ML252 inhibition.** (A) Exemplar records from Xenopus oocytes co-injected with mRNA expressing Kv7.3* or Kv7.3*[W265F] (homomeric channels), and treated with ML252 as indicated. (B) ML252 was applied between 0.1 μM and 100 μM, and currents normalized to control conditions to generate a concentration response (note: Kv7.3* data is re-plotted from Figure 1; dashed line). For the concentration response, currents were measured at +20 mV (n=6-8, data shown as mean ± SEM).


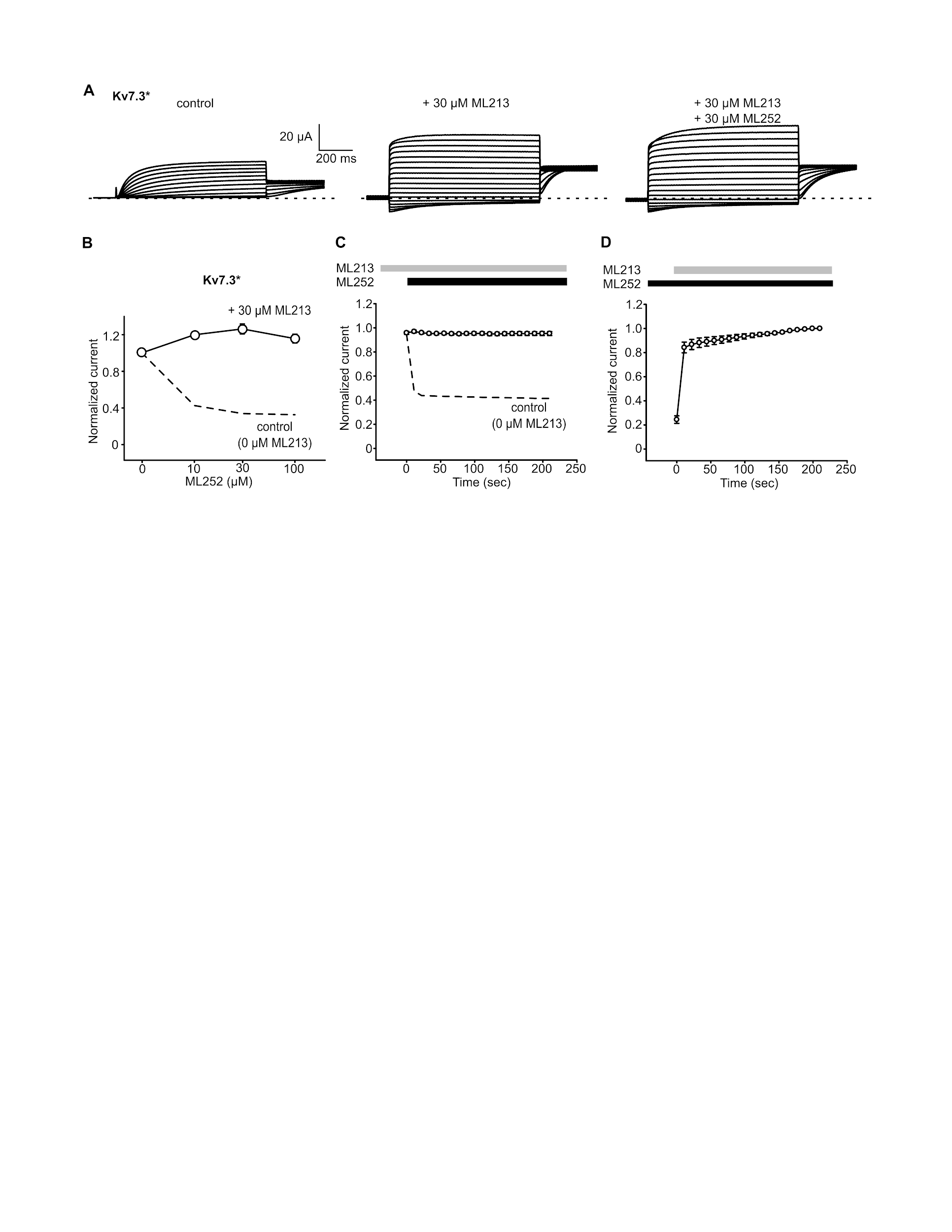


**Supplementary Figure 2:** **The pore-targeted activator ML213 prevents ML252 inhibition of Kv7.3*.** (A) Exemplar traces from a Xenopus oocyte expressing Kv7.3* homomeric channels, treated with indicated combinations of ML213 and ML252. Oocytes were held at -80 mV and pulsed between -120 mV and +40 mV. (B) ML252 concentration response (measured by inhibition of current at +20 mV) in the presence or absence of ML213 (note the dashed line for 0 μM ML213 is reproduced from Figure 1F)(n = 5-11). (C) Oocytes were pulsed from -80 to +40 mV for 500 ms at 0.1 Hz, first in 30 μM ML213, followed by 30 μM ML252 (in combination with 30 μM ML213) administration after the 1st pulse at time = 0 (n=8). The dashed line illustrates the onset of ML252 inhibition in 0 μM ML213. (D) With the same 0.1 Hz pulsing as in panel (C), oocytes were initially held in 30 μM ML252, followed by addition of 30 μM ML213 (in combination with ML252) after the 1st pulse at time = 0 (n=8).
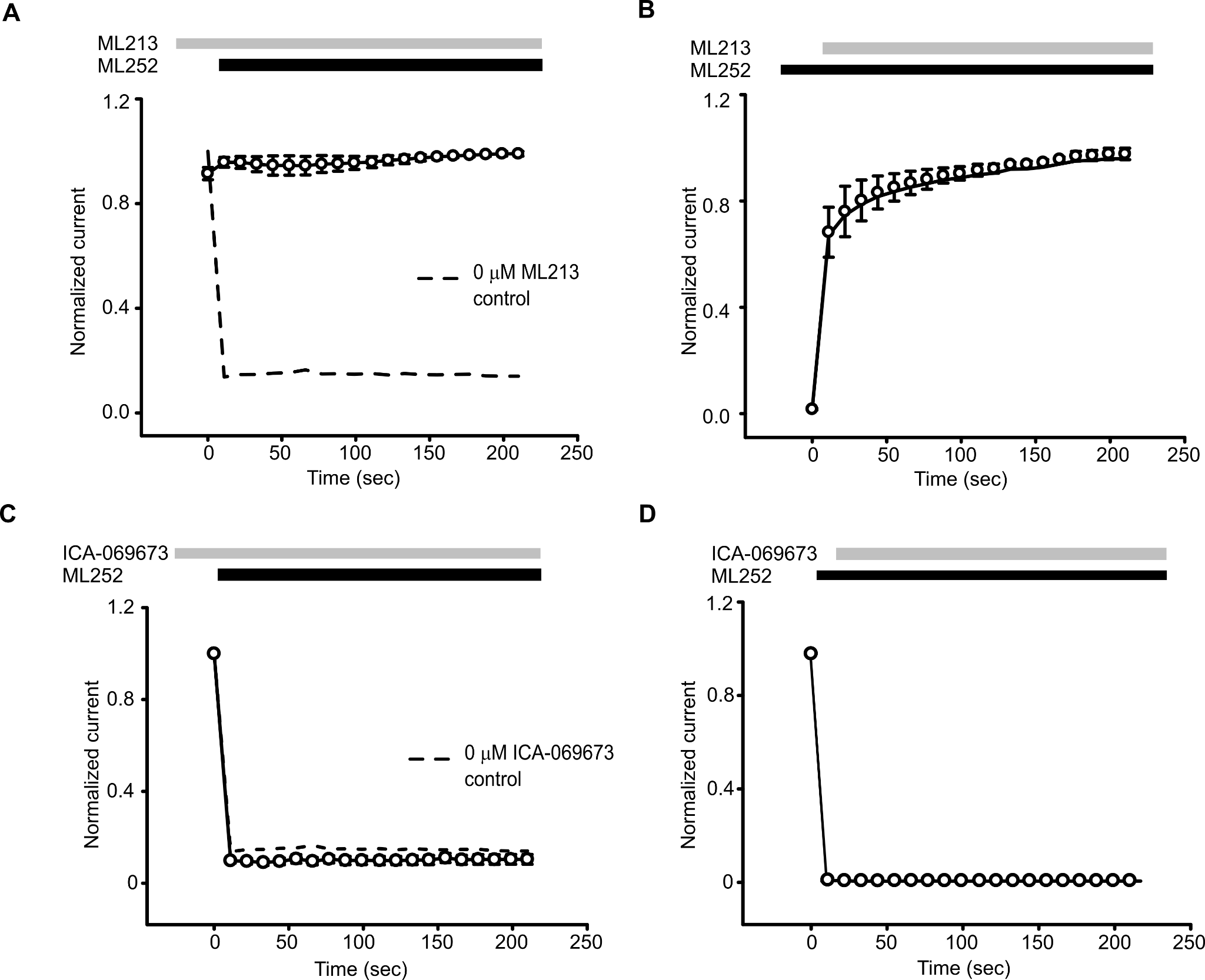


**Supplementary Figure 3:** **Distinct effects of pore- and VSD-targeted activators on ML252-mediated inhibition of Kv7.2.** (A, C) Kv7 activators ML213 or ICA-069673 (30 µM) were first applied alone, followed by a mixture of the activator plus 10 μM ML252. Oocytes were pulsed for 1 s at 0.1 Hz, from a holding potential of -80 mV to a test potential of +20 mV, and currents were normalized to the peak current recorded in each oocyte. Dashed lines illustrate ML252 inhibition in the absence of either activator. (B, D) Using the same 0.1 Hz pulse protocol as panels A,C, oocytes were first held in ML252 (10 µM) alone, followed by application (30 µM) of either ML213 (B) or ICA-069673 (D) in combination with ML252 (n=3-9).


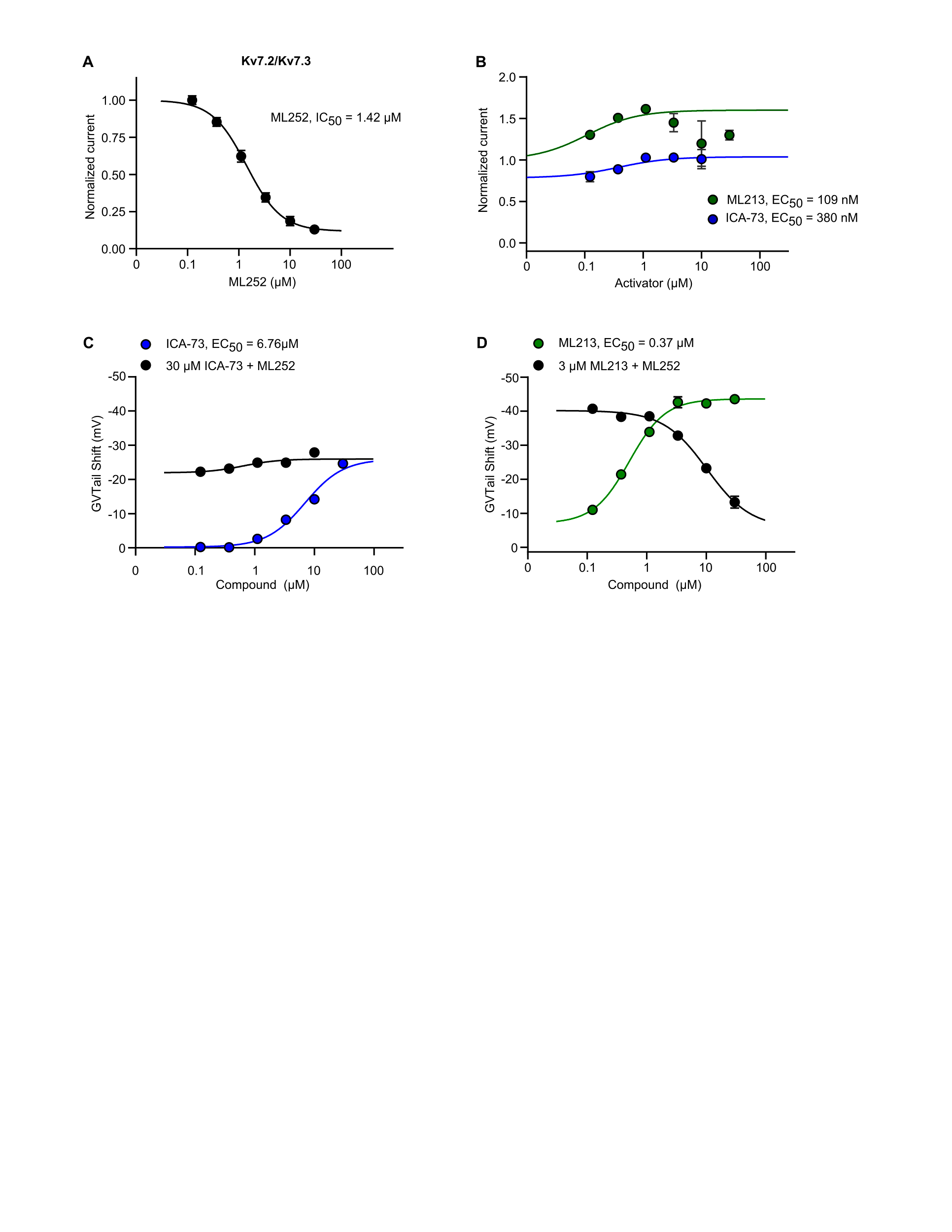


**Supplementary Figure 4:** **Functional interactions of ML252, ML213, and ICA-73 in Kv7.2/Kv7.3 heteromeric channels.** Concentration responses in all experimental conditions were determined using automated planar patch clamp of HEK293 cells stably expressing Kv7.2 and Kv7.3, as described in Figure 5. (A and B) Concentration response curves of normalized currents at +20 mV in response to ML252, ML213, or ICA-73. (C and D) Concentration response of drug mediated shifts of the V1/2 of activation, in the presence or absence of ML252. (C) ICA-73 causes a pronounced hyperpolarizing shift of activation V1/2 that is not influenced by ML252. (D) ML213 causes a pronounced hyperpolarizing shift of activation V1/2 that is attenuated by high concentraitons of ML252.


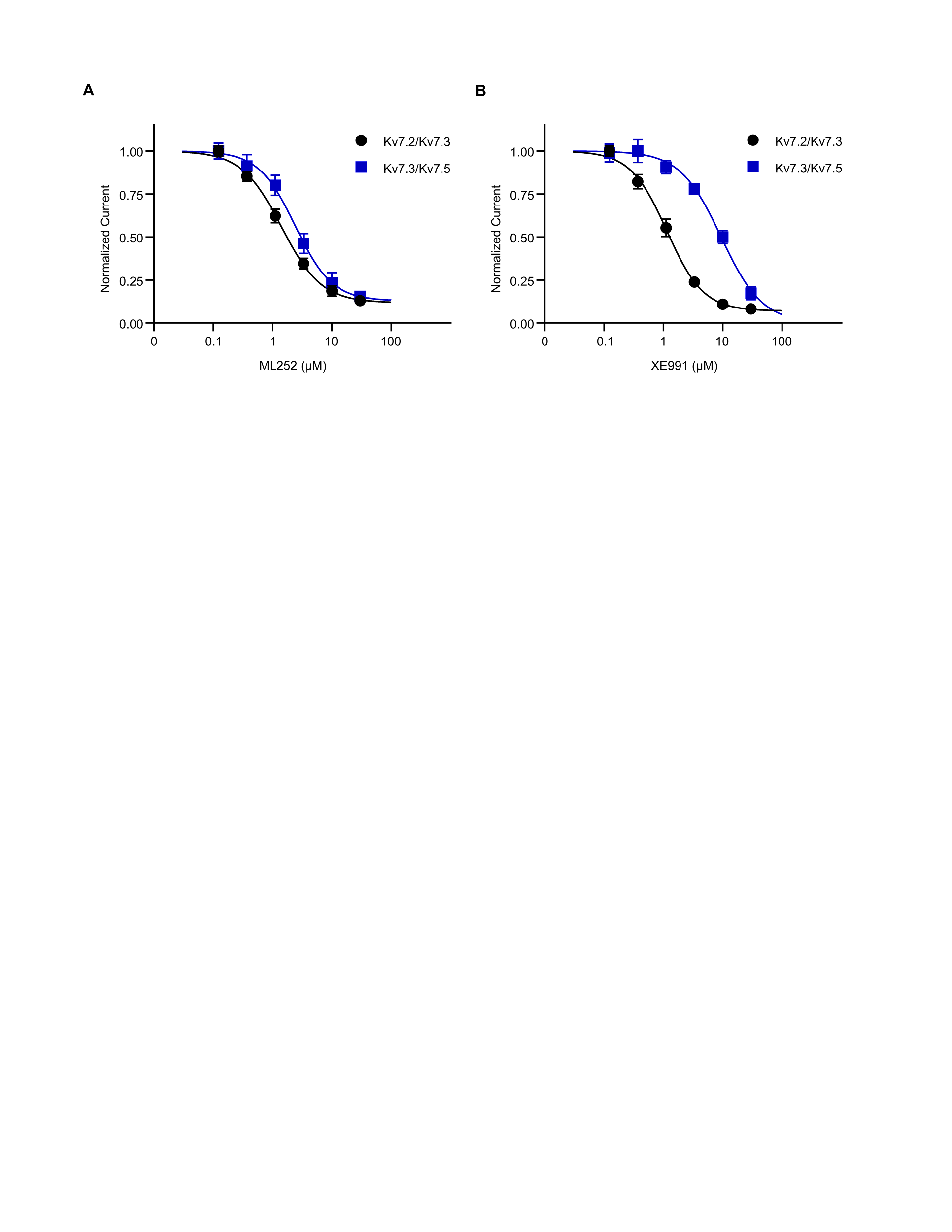


**Supplementary Figure 5. Automated patch clamp analysis of ML252 and XE991 mediated inhibition of Kv7.2/Kv7.3 and Kv7.3/Kv7.5 heteromeric channels.** Concentration responses in all experimental conditions were determined using automated planar patch clamp of HEK293 cells stably expressing Kv7.2 and Kv7.3, or Kv7.3 and Kv7.5. (A), Concentration responses to ML252 of Kv7.2/Kv7.3 (IC50=1.42 μM) and Kv7.3/Kv7.5 (IC50=2.43 μM). (B) Concentration responses to XE991 in Kv7.2/Kv7.3 (IC50=1.15 μM) and Kv7.3/Kv7.5 (IC50=9.42 μM). Currents (measured at 20 mV) were normalized to response in control conditions in each respective well containing up to 10 cells (n=4-8 wells).
